# 827. High KSHV Seroprevalence Among MSM with HIV Associated with Oral Intercourse and Methamphetamine Use in the Southern United States

**DOI:** 10.1093/ofid/ofab466.1023

**Published:** 2021-12-04

**Authors:** Sheena Knights, Maverick Salyards, Noelle Kendall, Susana Lazarte, Radhika Kainthla, Wendell Miley, Vickie A Marshall, Nazzarena Labo, Denise Whitby, Elizabeth Chiao, Ank E Nijhawan

**Affiliations:** 1 University of Texas Southwestern Medical Center, Dallas, TX; 2 UT Southwestern Medical Center, Dallas, TX; 3 UT Southwestern, Coto de Caza, CA; 4 Frederick National Laboratory for Cancer Research, Frederick, MD; 5 University of Texas MD Anderson Cancer Center, Houston, TX; 6 University of Texas Southwestern, Dallas, TX

## Abstract

**Background:**

Despite a decrease in Kaposi’s sarcoma (KS) cases in much of the US, the incidence of KS and associated mortality is increasing in specific subpopulations, particularly young, African American men in the South. To further understand this disparity, we sought to describe the seroprevalence and risk factors associated with Kaposi’s sarcoma herpesvirus (KSHV) among men who have sex with men (MSM) and transgender women (TGW) with HIV in Dallas, Texas.

**Methods:**

We enrolled MSM and TGW with HIV and without known KSHV-related disease from a large urban safety-net clinic in Dallas. Blood samples were collected from participants for IgG testing (K8.1 and ORF73), followed by KSHV PCR on blood and saliva samples for those with positive IgG results. We also collected demographics, sexual history, sexual practices, HIV history, substance use, and insurance status. Multivariate logistic regression modeling was performed to identify associations with KSHV seropositivity.

**Results:**

Of 159 participants, 110 (69.2%) were seropositive for KSHV. Seroprevalence varied by race/ethnicity, with 27/34 (79.4%) Hispanic, 27/37 (73.0%) white, and 54/84 (64.3%) black participants testing positive for KSHV IgG, though this difference was not statistically significant. 31/104 (29.8%) seropositive participants had detectable KSHV in saliva and 10/104 (9.6%) seropositive participants had detectable KSHV in blood. Risk factors independently associated with KSHV seropositivity include oral-anal sex (OR 4.02, 95% CI 1.89 – 8.54), oral-penile sex (OR 3.66, 95% CI 1.16 – 11.57), and methamphetamine use (OR 2.73, 95% CI 1.23 – 6.04). Current CD4 count, HIV viral load, history of intravenous drug use, tobacco or alcohol use were not associated with KSHV seropositivity.

Table 1. Patient Characteristics

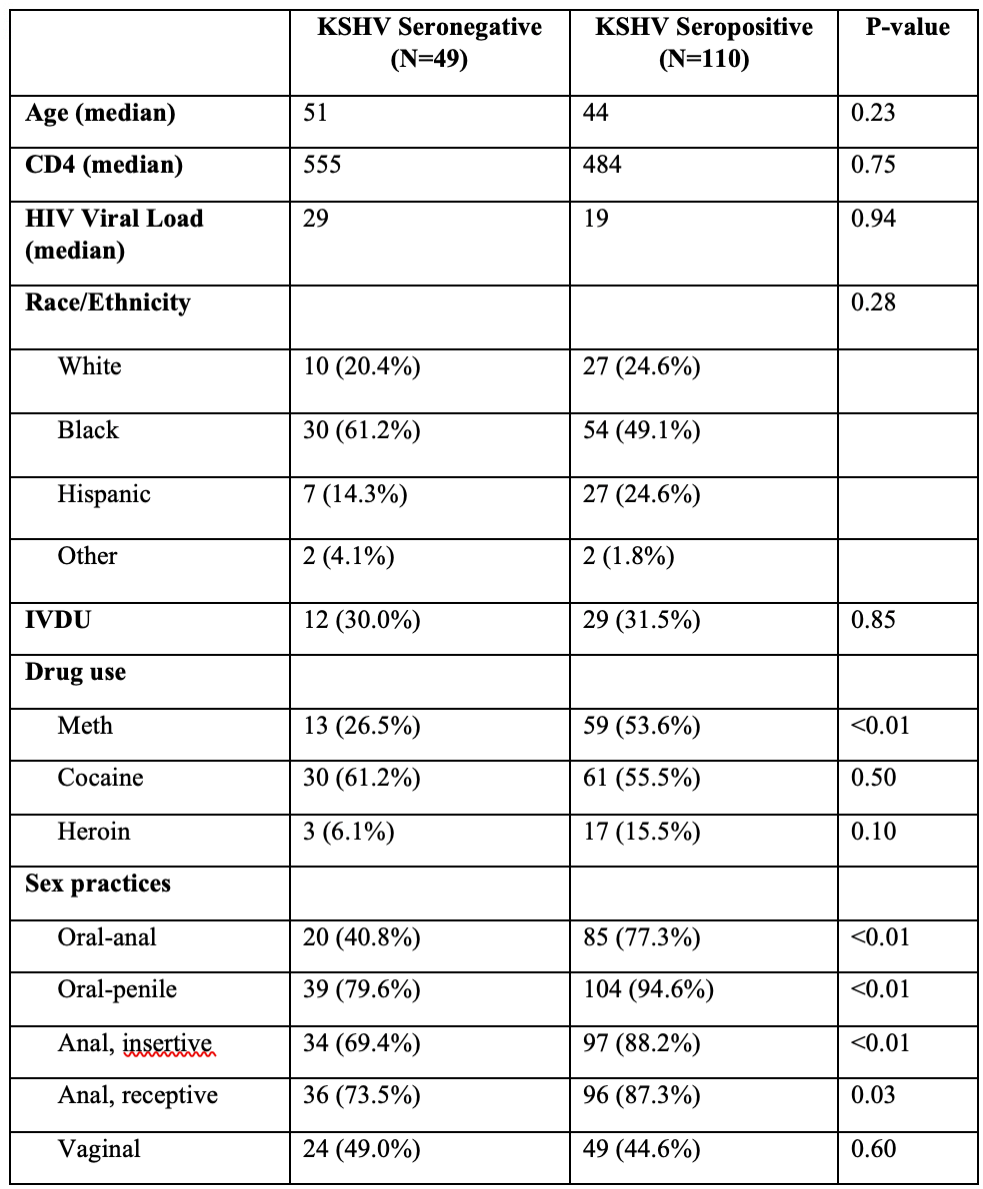

**Conclusion:**

We found that over two-thirds of MSM and TGW with HIV in Dallas are KSHV seropositive, which is relatively high compared to other studies of US MSM with HIV (30-70%). In our study, KSHV was more common among Hispanic and white individuals, and was associated with higher rates of oral sex and methamphetamine use. Differences in KSHV seroprevalence alone are unlikely to explain racial disparities in the incidence of KS. Further study is needed to better understand drivers of KSHV infection and KSHV-related diseases in highly impacted groups in the US.

**Disclosures:**

**All Authors**: No reported disclosures

